# Effect of a Low-Glycemic Load Diet Intervention on Maternal and Pregnancy Outcomes in Obese Pregnant Women

**DOI:** 10.3390/nu13030748

**Published:** 2021-02-26

**Authors:** Janina Goletzke, Jessica De Haene, Naomi E. Stotland, Elizabeth J. Murphy, Marcela Perez-Rodriguez, Janet C. King

**Affiliations:** 1 Department of Nutritional Sciences and Toxicology, University of California, Berkeley, CA 94720, USA; j.goletzke@fastmail.com; 2 Independent Nutrition Consultant, Dallas, TX 75035, USA; jessicadehaene@gmail.com; 3 Department of Obstetrics, Gynecology, and Reproductive Sciences, University of California San Francisco, San Francisco, CA 94110, USA; naomi.stotland@ucsf.edu; 4 Department of Medicine, Division of Endocrinology and Metabolism, San Francisco General Hospital, University of California, San Francisco, San Francisco, CA 94110, USA; Lisa.Murphy@ucsf.edu; 5 Research Coordinator, Mexican Institute of Social Security (IMSS), Mexico City 06720, Mexico; marxelapr@gmail.com

**Keywords:** glycemic load, obese women, pregnancy, glucose metabolism, dietary intervention

## Abstract

The increased prevalence of obese, pregnant women who have a higher risk of glucose intolerance warrants the need for nutritional interventions to improve maternal glucose homeostasis. In this study, the effect of a low-glycemic load (GL) (*n* = 28) was compared to a high-GL (*n* = 34) dietary intervention during the second half of pregnancy in obese women (body mass index (BMI) > 30 or a body fat >35%). Anthropometric and metabolic parameters were assessed at baseline (20 week) and at 28 and 34 weeks gestation. For the primary outcome 3h-glucose-iAUC (3h-incremental area under the curve), mean between-group differences were non-significant at every study timepoint (*p* = 0.6, 0.3, and 0.8 at 20, 28, and 34 weeks, respectively) and also assessing the mean change over the study period (*p* = 0.6). Furthermore, there was no statistically significant difference between the two intervention groups for any of the other examined outcomes (*p* ≥ 0.07). In the pooled cohort, there was no significant effect of dietary GL on any metabolic or anthropometric outcome (*p* ≥ 0.2). A post hoc analysis comparing the study women to a cohort of overweight or obese pregnant women who received only routine care showed that the non-study women were more likely to gain excess weight (*p* = 0.046) and to deliver large-for-gestational-age (LGA) (*p* = 0.01) or macrosomic (*p* = 0.006) infants. Thus, a low-GL diet consumed during the last half of pregnancy did not improve pregnancy outcomes in obese women, but in comparison to non-study women, dietary counseling reduced the risk of adverse outcomes.

## 1. Introduction

The prevalence of obesity in pregnancy is increasing worldwide and the health consequences are substantial for both the mother and her newborn. Obese pregnant women are more likely to have higher levels of insulin resistance, be at a greater risk to develop impaired glucose tolerance or gestational diabetes mellitus (GDM), and to experience complications, i.e., preeclampsia or deliver a macrosomic infant [1,2,3,4,5]. Of note, worldwide, and in the United States, ethnic disparities exist in pregnant women affected by maternal obesity, with Hispanic women having a higher prevalence than White women [6,7].

Normally, pregnancy involves a progressive increase in insulin resistance beginning mid-gestation that reduces maternal glucose utilization and, therefore, facilitates glucose uptake by the fetal-placental compartment to promote fetal growth [8,9,10]. These physiological adjustments may be enhanced in obese, pregnant women as elevated levels of insulin resistance preconception and in early gestation will increase the risk of glucose intolerance and fetal overgrowth in late gestation [11].

Studies in non-pregnant populations show that the type of dietary carbohydrate consumed influences glucose homeostasis and affects the risk of developing type 2 diabetes mellitus. The glycemic index (GI) is a method for ranking the postprandial glycemic response of carbohydrate-containing foods [12]. The glycemic load (GL) of a food is the product of the GI and the total available carbohydrate content in a given amount of food [12]. Several meta-analyses have associated a high GL diet with an increased risk of type 2 diabetes [13,14,15,16].

Studies of the effect of a low dietary GL on pregnancy outcomes are limited. But, a meta-analysis of randomized controlled trials of low-GI diets in pregnancy shows a reduction of the risk of developing hyperglycemia among high-risk women [17]. In a study comparing pregnancy outcomes in lean and obese women, we found that the obese women had a significantly greater postprandial glucose and insulin response to a high GI breakfast compared to a low glycemic meal [18]. Evidence from cohort studies, i.e., the Nurses’ Health Study, showed that a low fiber, high GL diet prior to pregnancy increased the risk for GDM by about 60% [19]. Furthermore, the risk of large-for-gestational age babies increased significantly when the lowest to highest quintile of GL was compared in 47,003 Danish women [20].

To further clarify the effects of a low versus high-GL diet during pregnancy, we compared the effect of a low-GL versus a higher-GL dietary intervention during the second half of pregnancy on maternal glucose metabolism and pregnancy outcomes in overweight and obese women. As this is a higher risk population, we specifically aimed to include Hispanic and African American pregnant women and women of low socioeconomic status. We hypothesized that the low-GL diet would be superior in achieving lower fasting and 3h-incremental area under the curve (3h-iAUC) values for glucose and insulin. We also compared the outcomes of our study participants to the outcomes of overweight and obese pregnant women attending the same antenatal clinic who received routine care without any dietary intervention.

## 2. Materials and Methods

### 2.1. Study Design and Population

Pregnant women with either a body mass index (BMI) ≥30 or a body fat >35% at study entry (20 weeks’ gestation), >18 to <42 years of age, with singleton pregnancies and no previous diagnosis of chronic disease, were recruited from two prenatal clinics affiliated with the University of California at San Francisco (UCSF) (i.e., the San Francisco General Hospital (SFGH) and Moffitt Hospital) between May 2005 and November 2009. Other entry criteria included willingness to follow a prescribed diet for the last half of pregnancy, agreement to participate in all measurements, plans to remain in the area until delivery, and the ability to understand and give informed consent in either English or Spanish. Women diagnosed previously with type 1 or 2 diabetes mellitus or gestational diabetes were excluded. Other exclusion criteria included the use of medications that affect metabolic parameters (e.g., thyroid hormones or steroid use), cigarette smoking, excessive drug or alcohol use, a moderately high level of physical activity (>90 min/week of activity with a physical activity level of >1.6), chronic hypertension (systolic blood pressure >130 mm Hg and/or diastolic blood pressure >90 mm Hg and/or use of antihypertensive medications), the presence of other chronic metabolic disease, such as cardiovascular disease, active thyroid disease, liver disease, pulmonary or psychiatric disorders, HIV, anemia (hematocrit < 30% and/or hemoglobin < 9.5 g/dL), any disorder requiring diet therapy (i.e., renal insufficiency), multiple gestation, or evidence of intrauterine growth retardation.

The protocol and consent form were reviewed and approved by the UCSF Committee of Human Research at UCSF. In light of the unavailability of published study data in obese pregnant women, we used data from a preliminary study measuring the 3 h glycemic response to a low and high glycemic load breakfast meal in 7 obese pregnant women to calculate the sample size. The average AUCs for glucose following the high and low glycemic meals were 242 ± 69 and 105 ± 38 mg/dL/h, respectively. Power calculations were based on a *t*-test with a 2-sided type I error rate of 5% and 80% power, and specified 20 subjects per treatment group to detect a difference of 50 mg/dL/h (3000 mg/dL/min). Mean between-group differences in iAUC were considerably smaller in our study, indicating insufficient power.

**Figure 1:**
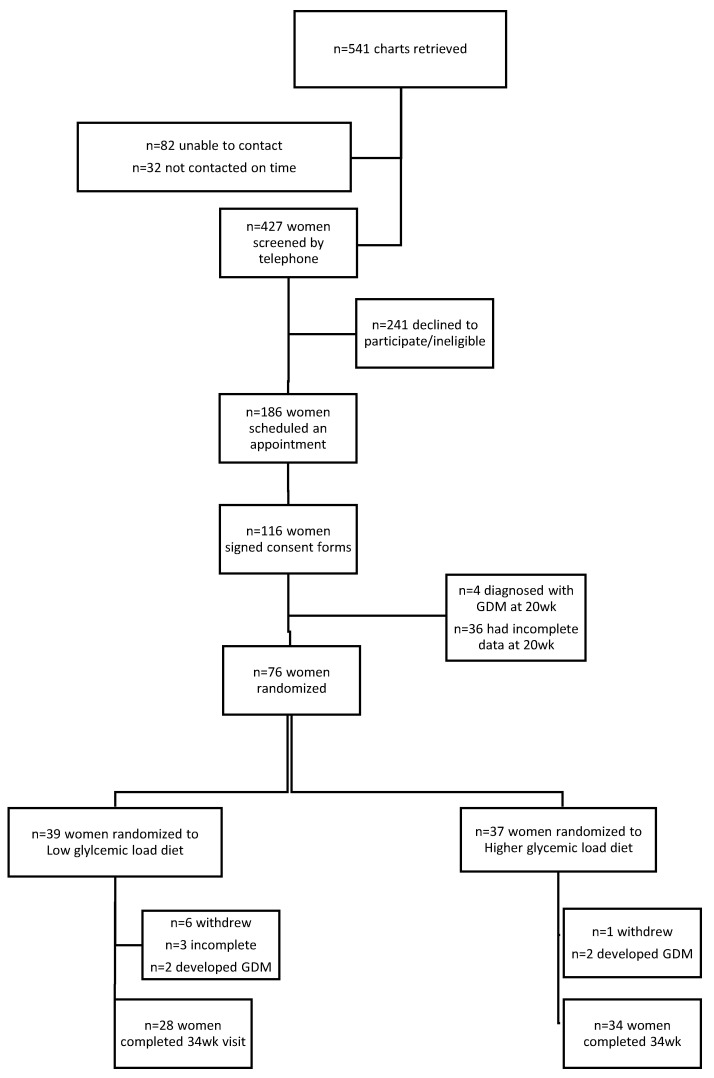
Flow chart participant recruitment and enrollment.

An initial phone screening was undertaken to determine eligibility for the study. Potentially eligible women were then interviewed in person. A staff member reviewed the consent form with all eligible and interested women. After signing the consent form, 116 women were scheduled for the first clinical visit. Thirty-six subjects failed to complete the first visit and four of the consented subjects were diagnosed with gestational diabetes mellitus (GDM) at the first visit. Thus, after the first visit, a total of 76 women were randomized to one of the two dietary treatments, the low glycemic load diet (LGL) (*n* = 39) or the moderately higher glycemic load diet (HGL) (*n* = 37). Of those women, two of the women in each group were diagnosed with GDM at 28 weeks and were dropped from the study. They received dietary counseling and medical care at SFGH. Women developing other pregnancy complications, i.e., pregnancy-induced hypertension, pre-eclampsia, persistent bleeding, or chronic infection, were retained in the study. Withdrawal from the study led to a total of 28 and 34 women completing the study in each group, respectively (Figure 1).

All clinical measurements were completed at the Clinical and Translational Research Center (CTRC) at SFGH. At 20 week gestation (baseline), the participants completed a health history questionnaire regarding previous pregnancies and deliveries, family history of chronic diseases, and current medications and health habits. Pre-pregnancy body weight was self-reported. Clinical appointments for body weight and composition measurements and for an oral glucose tolerance test (OGTT) were scheduled at 20, 28, and 34 weeks of gestation. Birth outcome data were obtained from the medical charts.

### 2.2. Dietary Intervention

Participants were randomized to a LGL- or a HGL-diet at entry into the study (20 weeks gestation). Educational materials and tools, graciously provided by Dr. Ebbeling, Department of Medicine, Children’s Hospital Boston, Boston, MA, USA were adapted to be consistent with the cultural food habits of Hispanic and African-American women in California. The visual materials included a plate divided into 3 parts for each food group (cereals and grains, proteins, and vegetables). Half of the LGL group plate had non-starchy vegetables (2 cups) and the other half was split into 2 parts: 85–113 g of protein (not restricted by fat content) and ½–3/4 cup of low-GI whole grain cereals (i.e., muesli, whole wheat pasta or corn tortillas). Low GI fruits, such as apples, were suggested for dessert. The HGL group’s plate was equally divided into three parts: one third moderately high GI cereals and grains (approximately 1 cup); one third low fat protein options (85–113 g), and one third starchy vegetables (i.e., mashed or baked potatoes and sweet potatoes) (1 cup). Higher-GI fruits such as watermelon were suggested for dessert. The women also received a suggested pantry list, cooking methods, and recipes. All women were advised to eat ad libitum, or until they were satisfied, as long as they maintained the specified distributions on their plates. The women received dietary counseling every two weeks and were given monthly food baskets with appropriate carbohydrate sources for their diet. The same nutritionist counseled the women about their diet pattern throughout the study. At the 20 week gestation interview, the nutritionist reviewed a 4-day diet record kept by each woman to determine the woman’s food preferences and eating pattern. Appropriate cooking methods were described and portion sizes were demonstrated using Nasco* food replicas, measuring cups, and spoons. At the end of this initial session, each woman received an individualized recommended dietary pattern, a list of foods allowed for each food group, pictures of the recommended distribution of food groups on a plate, and a grocery bag with appropriate carbohydrate or fat sources for her diet assignment. During the following week, follow-up phone calls were made to review the primary principles of the diet and to answer any questions. Three more bags of groceries were provided at 24, 28, and 32 weeks of gestation along with diet information. Women were contacted by phone at 22, 26, and 30 weeks to reinforce principles of their diet, answer questions, review the acceptance of foods in the grocery bag, and to modify the bag accordingly.

To monitor compliance with the dietary intervention, a nutritionist, other than the nutrition educator who did the dietary instructions, completed three to seven random 24-hr dietary recalls between 22 to 34 weeks of gestation using the Nutrition Data System for Research (NDS-R; Version 2010, Minneapolis, MN, USA). The nutrition educator reviewed the 24-h recalls. If any dietary deviations were noted, she then either phoned the women to provide further instructions and motivation to adhere to their diet plan or discussed it with them at their next clinic visit. The analysis of nutrient and food intakes was undertaken using the NDS-R software. Recalls reporting ≤900 kcal and ≥3500 kcal were considered invalid and not included in the average for a woman. Three women failed to provide at least three valid 24-h recalls; they were not included in the final analyses. As availability for the 24-h dietary recalls varied widely among participating women, the average of all 24-h recalls was calculated to determine a woman’s intake of nutrients and food groups during the whole study period.

### 2.3. Outcome Measurements

#### 2.3.1. Maternal Anthropometrics and Body Composition:

Body height measurements were taken using a wall stadiometer at 20, 28, and 34 weeks. Body weight was measured on a calibrated electronic scale. In addition, air plethysmography (Bod Pod, COSMED srl–Concord, CA, USA) was used to determine body density and volume. All of the measurements were done in duplicate; each measurement took approximately 1 min. The women were advised not to consume any food or drinks for 2 h prior to the measurements. Body mass and density values obtained from the BodPod measurement were entered into pregnancy-appropriate formulas to calculate body fat and fat-free mass [21].

#### 2.3.2. Oral Glucose Tolerance Test, Blood Sampling, and Analysis

Women completed a 3 h, 100 g oral glucose tolerance test (OGTT) at 20, 28, and 34 weeks gestation. They came to the CTRC (Clinical and Translational Research Center) at 8 AM after a 10 h fast (only plain water was allowed). Blood samples were collected from the antecubital vein using Becton–Dickinson (BD, Mississauga, ON, Canada) Insyte catheters into BD Vacutainers at fasting (baseline) and at 30, 60, 90, 120, and 180 min after the glucose load. Plasma and serum were separated by centrifugation (Sorvall RC-5C, Golden valley, MN, USA) at −4 °C for 10 min and stored at −80 °C until sample analysis was conducted. Plasma glucose levels were determined using the glucose-oxidase method with a glucose and lactate analyzer (YSI 2300 STAT Plus, Yellow Springs, OH, USA). Plasma insulin concentrations were analyzed using human radioimmunoassay kits (Linco Research Inc., St. Charles, MO, USA). All determinations were done in duplicate. The 3h-iAUC for glucose and insulin was calculated using the trapezoidal method ignoring the area beneath the fasting concentration. Insulin secretion was calculated using two methods: the homeostatic model assessment of insulin resistance (HOMA-IR) and the insulin sensitivity index (ISI). HOMA-IR, an index based on fasting glucose and insulin values, was calculated as follows: HOMA-IR = (fasting glucose (mg/dL) × fasting insulin (uU/mL)/405) [22]. This model assumes that normal individuals have an insulin resistance of 1. High HOMA-IR scores denote a low insulin sensitivity (i.e., increased insulin resistance). The insulin sensitivity index (ISI) was calculated using the Matsuda–DeFronzo equation [23]: ISI = 10,000/square root of ([fasting glucose × fasting insulin] × [mean post load glucose × mean post load insulin]). Furthermore, the McAuley-Index was calculated as an alternative surrogate marker measuring insulin resistance (McAuley = exp (2.63–0.28 ln [fasting serum insulin] - ln [fasting serum triglycerides]) [24]. All calculations were done with plasma insulin concentrations expressed as μU/mL and plasma glucose and serum triglycerides concentrations expressed as mmol/L.

#### 2.3.3. Pregnancy Outcomes

Data regarding birth weight, length, head circumference, gestational age, gender, and type of delivery were obtained from the newborn’s medical chart. Percentiles for weight, length, and head circumference were calculated using the CDC growth charts, 2008 (www.cdc.gov/growthcharts, accessed on November 2020). Ponderal index was calculated as the birth weight (kg) divided by length(m)^3^. Infants with a birth weight and length ≥10th and ≤90th percentile were categorized as appropriate-for gestational age (AGA). Large-for-gestational-age (LGA) is defined as birth weight and length ˃90th percentile. Macrosomia was defined as a birth weight >4000 g.

#### 2.3.4. Data from the Control Cohort

Anonymized data on gestational weight gain, gestational age, and birth weight data were retrieved for eligible women using the same inclusion and exclusion criteria applied as for study participants receiving care at San Francisco General Hospital between August 2006 and August 2009. Women who had developed gestational diabetes mellitus (GMD) at some point during pregnancy were excluded.

### 2.4. Statistical Analyses

The study population characteristics and dietary intake data for continuous variables are presented as means and standard deviations or medians and quartiles (25th, 75th percentile) were calculated for normally and not normally distributed continuous variables, respectively; total numbers (n) and percentages were presented for categorical variables. Participant characteristics and dietary intake data are presented separately for the low-GL and high-GL group (LGL and HGL). They were compared using analysis of variance (ANOVA) for normally distributed continuous variables, the Kruskal–Wallis test for non-normally distributed continuous variables and the Chi-square test for categorical variables.

To examine an effect of the nutritional intervention for each group, mean changes in the respective outcome variables were calculated (i.e., parameter at week 34 minus parameter at week 20). Also, mean between-group differences were calculated and presented as means (95% confidence intervals) and the differences were assessed using Student’s *t*-test.

To increase statistical power, in addition to the described intervention group comparisons, data from both intervention groups were pooled to assess a continuous effect of glycemic load on maternal metabolic parameters. Linear mixed-effect regression models (PROC MIXED in Statistical Analysis System (SAS)), including both random and fixed effects, were computed. Non-normally distributed parameters were log-transformed to achieve normal distribution. The GL was energy-adjusted using the residual method and all models were adjusted for the effect of time (defined as duration in weeks between the baseline and final visit), intervention group, and BMI at study baseline. These analyses were only conducted for repeatedly collected maternal parameters during pregnancy and not for birth outcomes.

For the additional study aim to compare the pooled intervention cohort to obese pregnant women receiving routine antenatal care only, propensity score matching was applied to ensure equal distribution of possible confounding variables among those women receiving a nutritional intervention and those who did not [25]. Propensity scores were determined using logistic regression models in all participants with information on gestational weight gain during the study period and birthweight data. Propensity score matching (1:1) was performed based on maternal age, study baseline BMI, parity, family history of diabetes. Calipers of width equal to 0.4 standard deviations of the logit of the propensity score, respectively, were calculated separately per subgroup. Descriptive statistics indicated that measured confounders, i.e., maternal age and BMI, were well balanced between both groups after matching (Supplementary Materials Table S1) [26]. Matched groups were compared using paired *t*-tests to account for the clustered structure [25].

All analyses were carried out by using SAS software (version 9.4; SAS Institute) and were performed with a significance level at *p* < 0.05.

## 3. Results

A total of 28 and 34 women were randomized into the LGL- and HGL-groups, respectively (Table 1). The characteristics of the participants did not differ between the two intervention groups. About two-thirds of the women were Hispanic and about half had a family history of diabetes.

Table 2 presents the mean dietary intake during the study (gestational week 22–34) for the LGL- and HGL-groups. The energy intake of the LGL women was significantly lower than that of the HGL-group (*p* < 0.0001). The percentage of energy intake from carbohydrates, fat, and protein did not differ between the two intervention groups (*p* ≥ 0.3). As per the study’s aim, the dietary GL was significantly lower in the LGL group compared to the HGL group (*p* < 0.0001 for GL (g) and *p* = 0.009 for GL, g/1000 kcal). Also, women in the LGL group had a significantly lower dietary GI (*p* = 0.01) and a lower intake of total sugars (*p* = 0.002).

Table 3 presents the effect of the dietary intervention on maternal metabolic and anthropometric outcomes as well as pregnancy outcomes. Glucose measurements did not differ between the two groups at any study timepoint. For insulin, the only statistical trend for a difference between groups observed was at gestational week 28, when the 3h-insulin-iAUCs were higher for women in the LGL compared to the HGL group (*p* = 0.07). Among the examined metabolic indices, at gestational week 28, women in the LGL-group tended to have a lower Matsuda Index compared to the women in the HGL-group (*p* = 0.07). A higher Matsuda Index reflects an increased rate of postprandial plasma glucose disappearance. There were no differences between the two groups in the gestational weight gain or in the percentage of maternal fat mass at any study timepoint. Also, the measured pregnancy outcomes did not differ between the two intervention groups.

Since the effects of the dietary intervention between the two intervention groups were limited, the effects of the reported dietary GL during the study on metabolic and anthropometric outcomes were determined in a pooled study sample of all 62 women (Table 4). No statistically significant effect of dietary GL was observed for any metabolic and anthropometric outcomes (*p* ≥ 0.2).

Finally, the outcomes of women participating in the nutritional intervention study were compared with those receiving routine care only (*n* = 47 in both groups) using propensity score matched pairs (see Supplementary Materials Table S1 for distribution of the variables considered for matching) (Table 5). While there were no significant differences between gestational weight gain, birthweight, birth length or gestational age at birth (*p* ≥ 0.2) in the study and control groups, the percentage of excess weight gain (*p* = 0.046), large for gestational age (LGA) neonates (*p* = 0.01), and macrosomia (*p* = 0.006) was significantly higher among the women who only received routine care (Table 5).

## 4. Discussion

The effects of a low-GL intervention, consisting of both dietary counselling and monthly food boxes, during the second half of pregnancy on maternal metabolic and anthropometric measurements and pregnancy outcomes were determined in this study. No differences were observed for any of the outcomes.

Unlike most other dietary intervention studies, this study was unique in that it provided both repeated dietary counselling and a monthly food box over a 14-week period during the last half of pregnancy. The overall difference in GL was 6.7 g/1000 kcal, which is comparable to other studies that only achieved modest differences between intervention groups [28,29]. This moderate difference in the GL between the groups probably contributed to the lack of differences in study outcomes. However, it should also be noted that the low-GL-diet group had significantly lower energy and added sugar intakes, suggesting a potentially overall healthier diet. Also, the dietary GI only differed by 2.2 units, which reflects the study’s primary objective to modify the carbohydrate intake and, thereby, achieve a difference in GL. The GI is by definition the best parameter to estimate glycemic responses [12] and the small difference in dietary GI between study groups might have prevented differences due to the dietary intervention.

Unlike most previous studies of the effects of low/high-GI/GL diets in pregnancy that have been undertaken concerning women diagnosed with GDM or at high risk for GDM [15], this study focused on obese pregnant with normal glucose tolerance at 20 week gestation. To the best of our knowledge, only one other study evaluated a GL intervention in overweight/obese pregnant women [17]. In that study of comparable size to ours (*n* = 46), Rhodes and co-workers studied the effect of a LGL versus a low fat-diet on weight gain in overweight and obese pregnant women [30]: Despite a slightly longer study duration (18–23 weeks) compared to our 14-week study and a GL difference of 12.8 g/1000 kcal between study arms, no changes in fasting blood glucose or insulin concentrations nor in birth weight *Z* scores or infant adiposity measures were observed. However, women in the low-GL group had smaller increases in serum triglycerides and total cholesterol and a greater decrease in C-reactive protein during the study [30].

Women of minority ethnic backgrounds and low socioeconomic status were targeted in our study and represent the majority of our study population. Indeed, 74% of the women enrolled in our study were participants in WIC, the Special Nutrition Program for Women, Infants, and Children offered by the Food and Nutrition Service (FNS) in the U.S. Department of Agriculture. This program is designed for low-income pregnant, postpartum, and breastfeeding women, infants, and children up to the age of five years who are at nutritional risk. Through the program, participants are provided supplemental nutritious foods, nutrition education, and referrals for health care [31].

Unexpectedly, in the present study, women in the LGL-group tended to have higher 3h-insulin-iAUCs and lower Matsuda Indices at gestational week 28 compared to women in the HGL-group. Higher Matsuda-indices indicate a more beneficial metabolic state [23], and since this index includes postprandial values, one would expect lower 3h-insulin-iAUCs as well. In a previous study of non-obese pregnant women, consumption of a high-fiber or low glycemic diet was associated with a lower insulin response to a meal [32]. Other pregnancy studies of low GI/GL diets that assessed fasting insulin measures showed lower insulin measures with a low GI/GL in most [30,33] but not all [28] of the studies. The higher insulin iAUCs in our LGL women at 28 week needs to be interpreted with caution, as our dietary intake data reflect average intakes throughout the 14 week study period; no specific intake data are available for gestational week 28 when the insulin iAUCs were higher in the LGL group. It is hence difficult to find a possible explanation for this unexpected result.

The additional comparison of the study women to a cohort of obese pregnant women receiving routine care in the same antenatal clinic demonstrates the potential benefits of nutritional counselling during pregnancy. There were no differences in gestational weight gain and birth weight between the study and routine care participants. However, the proportion of women with excessive weight gain and LGA or macrosomic neonates was significantly higher in the routine care group. This demonstrates the importance of individualized nutritional counselling during pregnancy. Although the women in the routine care cohort were not randomized, by using the statistical approach of propensity score matching, a quasi-randomized study design was achieved and key maternal characteristics were equally distributed [25,26]. Even though some dietary counseling is part of the routine prenatal care, our results show an additional benefit when food boxes containing components of a healthy diet that could help manage weight gain and prevent macrosomia are provided. A future study that includes a randomized control group is needed to confirm the potential advantages of dietary interventions during pregnancy.

The repeated detailed assessment of maternal glucose and insulin metabolism is a strength of this study. However, the findings are limited by the high drop-out rate and by socioeconomic barriers to adhere to the dietary intervention. This was due, in part, to a study cohort that consisted primarily of Hispanic women with a low socioeconomic status. Personal daily family obligations and a general low interest in participating in a research study challenged their ability to be fully compliant with the study intervention. However, we chose to target those women because of the high prevalence of complications among these women and the limited information regarding dietary habits during pregnancy in this group. Unfortunately, the low compliance with the 24 h-dietary recalls, prevented us from determining the dietary intakes at each study timepoint, i.e., the dietary changes between gestational week 22 and 28 or 28 and 34. Also, evaluating the mean dietary data over the entire 12 weeks study period may have obviated any differences between the intervention groups at the three study time points.

## 5. Conclusions

This preliminary study failed to show an effect of a low -GL diet on maternal anthropometric and metabolic measures in overweight and obese pregnant women. Larger studies with greater differences in GI/GL between study groups are needed to better understand the impact of low-GI/GL diets on pregnancy outcomes. However, the results show that the provision of detailed dietary counselling to obese pregnant women reduced the risk of excess gestational weight gain, large-for-gestational age infants, and macrosomia.

## Figures and Tables

**Table 1:** Participant characteristics by intervention group.

	Low-GL Group (*n* = 28)		Higher GL Group (*n* = 34)		*p* for Difference ^1^
	*n*	Mean (SD)/Median (25th, 75h Percentile)	*n*	Mean (SD)/Median (25th, 75h Percentile)	
Age, years	28	29.3 (5.20)	34	28.3 (6.00)	0.5
BMI study baseline, kg/m^2^	28	33.1 (6.26)	34	32.2 (4.13)	0.5
Gravida	28	2 (1.5,4)	34	3 (2,4)	0.7
Parity	21	2 (1,2)	29	1 (1,2)	0.2
Ethnicity *n* (%)	28		34		0.2
Hispanic		22 (78.6%)		22 (64.7%)	
African American		3 (10.7%)		8 (23.5%)	
Caucasian		0 (0%)		3 (8.8%)	
Asian		0 (0%)		0 (0%)	
Hawaiian/Pacific Islanders		1 (3.6%)		0 (0%)	
Mixed ethnicity		2 (7.1%)		1 (2.9%)	
Family history of diabetes, *n* (%)	28	16 (57.1%)	34	15 (44.1%)	0.4

^1^ Analysis of variance (ANOVA) for normally distributed continuous variables, Kruskal–Wallis test for not normally distributed continuous variables and Chi-square test for categorical variables. BMI: body mass index.

**Table 2:** Participants’ dietary intake by intervention group ^1.^

	Low-GL Group (*n* = 28) Mean (SD)	Higher GL Group (*n* = 34) Mean (SD)	*p* for Difference ^2^

Energy, kcal	1460 (208)	1817 (361)	<0.0001
Carbohydrate, en% ^3^	53.3 (6.14)	55.1 (6.42)	0.3
Fat, en% ^3^	27.2 (6.08)	26.3 (5.90)	0.5
Protein, en% ^3^	19.2 (2.66)	18.5 (3.52)	0.4
Total sugars, g	88.8 (21.0)	113.0 (34.7)	0.002
Total dietary fiber, g	23.6 (8.05)	22.4 (6.43)	0.5
Soluble fiber, g	6.70 (2.50)	6.46 (2.22)	0.7
Insoluble fiber, g	16.9 (6.05)	15.9 (4.87)	0.5
Total whole grain, g	2.25 (1.36)	2.48 (1.83)	0.6
Dietary GI	53.5 (3.58)	55.7 (3.04)	0.01
Dietary GL (g)	95.1 (15.9)	130.9 (31.8)	<0.0001
GL, (g/1000 kcal)	65.4 (8.14)	72.1 (10.8)	0.009

^1^ mean intake between gestational week 22–34. ^2.^ ANOVA for normally distributed continuous variables and Kruskal-Wallis test for not normally distributed continuous variables^. 3.^ percentage of energy from respective macronutrient. GI: glycemic index; GL: glycemic load.

**Table 3:** Maternal metabolic parameters and pregnancy outcomes by dietary intervention group (*n* = 62).

	Low-GL Group		Higher-GL Group		Mean between-Group Difference (95% CI) ^1^	*p*
	*n*	Mean (SD)/Median (25th, 75h Percentile)	*n*	Mean (SD)/Median (25th, 75h Percentile)		
**Maternal metabolic parameters**						
**Fasting glucose** (mg/dL)						
gw 20	27	79.4 (5.50)	33	81.1 (5.50)	1.67 (−4.53; 1.19)	0.2
gw 28	25	81.1 (7.89)	34	82.7 (6.66)	−1.66 (−5.46; 2.14)	0.4
gw 34	25	81.2 (7.29)	34	82.5 (8.15)	−1.31 (−5.42; 2.81)	0.5
mean change	25	1.90 (−2.10; 5.80)	33	1.2 (−4.0; 3.8)	0.44 (−3.37; 4.24)	0.5
**Glucose 3h-iAUC** (mg/dL/min)						
gw 20	27	5904 (4101; 8358)	33	6551 (4899; 8712)	−376 (−1792; 1040)	0.6
gw 28	25	7239 (6201; 8984)	32	6394 (5022; 8533)	752 (−418; 1923)	0.3
gw 34	25	7161 (5703; 9933)	33	8051 (6002; 10266)	−148 (−1685; 1389)	0.8
mean change	25	2037 (647; 3108)	32	1232 (−467; 2552)	482 (−1152; 2120)	0.6
**Fasting Insulin** (μU/mL)						
gw 20	27	14.6 (8.1; 20.2)	33	13.1 (8.3; 18.5)	1.64 (−2.37; 5.66)	0.5
gw 28	25	18.2 (12.1; 22.2)	34	14.5 (10.0; 20.3)	2.83 (−2.26; 7.92)	0.16
gw 34	25	20.6 (14.5; 25.1)	34	16.0 (12.5; 23.6)	0.91 (−6.12; 7.94)	0.3
mean change	25	4.4 (1.4; 11.3)	33	4.4 (0.2; 6.2)	−0.48 (−6.14; 5.18)	0.7
**Insulin 3h-iAUC** (μU/mL/min)						
gw 20	27	10421 (6846; 19130)	33	10925 (8523; 13941)	−545 (−6187; 5096)	0.8
gw 28	25	15972 (8865; 19245)	33	10269 (8910; 13994)	3893 (−357; 8143)	0.07
gw 34	25	17828 (12360; 26306)	33	14178 (10829; 18116)	5346 (−4105; 14796)	0.3
mean change	25	6137 (677; 13281)	32	4167 (933; 6097)	705 (−569; 14266)	0.1
**HOMA-IR** **^2^**						
gw 20	27	2.98 (1.49; 3.96)	34	2.61 (1.42; 3.71)	0.36 (−0.53; 1.25)	0.5
gw 28	28	3.18 (2.07; 4.54)	34	2.92 (1.93; 4.66)	0.08 (−1.11; 1.26)	0.8
gw 34	28	3.63 (2.32; 5.05)	34	3.27 (2.50; 4.85)	−0.37 (−2.02; 1.28)	1.0
mean change	27	0.73 (−0.17; 2.34)	34	1.09 (0.16; 1.60)	−0.59 (−2.00; 0.82)	0.6
**Matsuda Index** **^3^**						
gw 20	27	2.82 (2.04; 5.49)	33	3.22 (2.40; 4.33)	0.24 (−0.83; 1.31)	0.9
gw 28	25	2.17 (1.80; 3.54)	33	2.84 (2.22; 3.85)	−0.34 (−1.11; 0.43)	0.07
gw 34	25	1.78 (1.54; 2.97)	33	1.75 (2.30; 2.97)	0.20 (−0.81; 0.41)	0.3
mean change	25	−1.33 (−2.95; −0.43)	32	−1.02 (−1.37; −0.42)	−0.64 (−1.49; 0.21)	0.3
**McAuley-Index** **^4^**						
gw 20	27	1.49 (0.39)	33	1.57 (0.34)	−0.08 (−0.27; 0.11)	0.4
gw 28	25	1.32 (0.40)	34	1.40 (0.24)	−0.08 (−0.25; 0.08)	0.3
gw 34	25	1.22 (0.30)	34	1.28 (0.25)	−0.06 (−0.20; 0.09)	0.4
mean change	25	−0.28 (−0.42; −0.13)	33	−0.24 (−0.41; −0.12)	0.02 (−0.10; 0.15)	0.9
**Maternal anthropometric parameters**						
**Gestational weight gain (between gw 20 and gw34)** (kg)						
	26	5.65 (3.10; 7.50)	33	5.67 (3.60; 8.55)	−1.10 (−2.89; 0.68)	0.4
**Excess gestational weight gain**, n (%) ^5^						
	26	18 (69.2%)	33	24 (72.7%)	-	0.8
**Maternal fat mass (%)**						
gw 20	28	39.57 (6.62)	34	40.04 (5.12)	−0.47 (−3.45; 2.51)	0.8
gw 28	27	39.57 (5.76)	33	39.26 (4.97)	0.31 (−2.46; 3.08)	0.8
gw 34	26	38.88 (6.75)	33	38.55 (4.54)	0.33 (−2.62; 3.28)	0.8
mean change	26	−1.70 (−2.71; 0.53)	33	−1.26 (−2.84; 0.80)	0.27 (−1.09; 1.63)	0.7
						
**Pregnancy outcomes**						
**Gender** (female)						
	28	15 (53.6%)	33	17 (51.5%)	-	0.9
**Gestational age** (week)						
	28	38.7 (1.65)	33	39.1 (1.43)	−0.39 (−1.18; 0.40)	0.3
**Birth weight** (g)						
	28	3383 (430)	33	3370 (458)	12.97 (−216; 242)	0.9
**Birth Length** (cm)						
	28	50.65 (2.14)	32	51.30 (1.96)	−0.66 (−1.72; 0.41)	0.2
**Head circumference** (cm)						
	22	34.7 (1.30)	26	34.3 (1.88)	0.45 (−0.51; 1.40)	0.3
**Appropriate for gestational age**, *n* (%)						
	28	17 (60.7%)	33	27 (81.8%)	-	0.2
**Cesarean section**, n (%)						
	28	5 (17.9%)	30	8 (26.7%)	-	0.4
**Pregnancy complications**, *n* (%)						
	26	4 (15.4%)	27	5 (18.5%)	-	0.5

^1^ Low glycemic load diet (LGL) minus high glycemic load diet (HGL) group; note that mean differences were only calculated for continuous variables. ^2^ Homeostatic model assessment of insulin resistance (HOMAIR) = (fasting glucose (mg/dL) × fasting insulin (uU/mL)/405) [22]. This model assumes that normal individuals have an insulin resistance of 1. High HOMAIR scores denote a low insulin sensitivity (i.e., increased insulin resistance). ^3^ Matsuda-Index: the insulin sensitivity index (ISI) was calculated using the Matsuda-DeFronzo equation: ISI = 10,000/Square root of ([fasting glucose × fasting insulin] × [mean post load glucose × mean post load insulin]) [23]. ^4^ McAuley-Index = exp (2.63–0.28 ln [fasting serum insulin] - ln [fasting serum triglycerides]) [24]. ^5^ according to Institute of Medicine guidelines: recommended upper range of weight gain in the second and third trimester: 0.6 lb/week/0.272 kg/week [27]. Abbreviations: CI, confidence interval; iAUC, incremental area under the curve.

**Table 4:** Results for the linear mixed effects regression models (proc mixed) for the association between dietary glycemic load and maternal anthropometric and metabolic parameters during pregnancy (gestational week 20–34) in the pooled cohort (*n* = 62).

	ß	95% CI	*p*

**Maternal metabolic measurements**			
Fasting glucose (mg/dL)	0.6867	−0.6236; 1.9970	0.3
Glucose 3h-iAUC	155.64	−401.37; 712.65	0.6
Fasting Insulin (μU/mL) ^1^	−0.00539	−0.1161; 0.1053	0.9
Insulin 3h-iAUC ^1^	391.99	−2111.96; 2895.93	0.8
HOMA-IR ^1^	0.002501	−0.1183; 0.1233	0.9
Matsuda Index ×	−0.01184	−0.1292; 0.1055	0.8
McAuley-Index	0.02113	−0.04718; 0.08943	0.5
**Maternal anthropometric measurements**			
Weight (kg) ^1^	0.01105	−0.01075; 0.03285	0.4
Fat mass (%) ^2^	−0.7322	−1.6551; 0.1924	0.2

Models contain a random statement with an unstructured covariance structure. Model a contains time (defined as duration in weeks between the baseline and final visit), mean glycemic load during the study (adjusted for energy using the residual method), fixed effects of intervention group, and BMI at study baseline. ^1^ logarithmized to achieve normal distribution. ^2^ measured in the Bod Pod and calculated using the Van Raiij formula.

**Table 5:** Pregnancy outcomes comparing those women participating in the nutritional intervention study (combined) to propensity-score matched controls.

	Study Participants (*n* = 47)	Routine Group (*n* = 47)	*p* for Difference ^1^
	Mean (SD)/Median (25th, 75h Percentile)	Mean (SD)/Median (25th, 75h Percentile)	
Gestational weight gain (between gw 20 and gw34), kg	5.69 (3.30, 7.80)	6.55 (4.50, 9.00)	0.2
Gestational weight gain (between gw 20 and gw34), kg/week	0.41 (0.24, 0.56)	0.47 (0.32, 0.64)	0.2
Excess gestational weight gain ^2^, n (%)	31 (66.0%)	41 (87.2%)	0.046
Gestational age, weeks	39.0 (1.40)	39.2 (1.66)	0.5
Birthweight, g	3390 (440)	3374 (523)	0.4
Macrosomia, *n* (%)	0 (0%)	7 (14.9%)	0.006
LGA, *n* (%)	1 (2.1%)	6 (12.8%)	0.01

^1^ logarithmized to achieve normal distribution. ^2^ according to IOM guidelines: recommended upper range of weight gain in the second and third trimester: 0.6 lb/week/0.272 kg/week [27].

## Supplementary Materials

The following are available online at https://www.mdpi.com/2072-6643/13/3/748/s1, Table S1: Distribution of the variables considered for propensity score calculation.
