# Supplementary material for: Effect of a Low-Glycemic Load Diet Intervention on Maternal and Pregnancy Outcomes in Obese Pregnant Women
Source: Nutrients. 2021 Feb 26;13(3):748. doi: 10.3390/nu13030748 (PMC7996780; doi:10.3390/nu13030748)
Supplement: Supplementary file 1 [file nutrients-13-00748-s001.pdf]

Supplemental Table 1: Distribution of the variables considered for propensity score calculation

	**Study participants** n=47 mean (SD)	**controls** n=47 mean (SD)	**p** for difference*
**Age,** years	29.6 (5.21)	29.3 (4.88)	0.6
**BMI study baseline**, kg/m^2	33.2 (5.52)	32.4 (4.49)	0.4
**Parity**	1 (1, 2)	1 (1, 2)	0.9
**Family history of diabetes,** n (%)	25 (53.2%)	27 (57.5%)	0.7

* paired t-test
